# Muscle and Fat Composition in OSA: A CT-Based Study

**DOI:** 10.3390/jcm14134647

**Published:** 2025-07-01

**Authors:** Hatice Beyazal Polat, Songül Özyurt, Mustafa Taştan, Fatma Beyazal Çeliker, Mehmet Beyazal, Ünal Şahin, Abdülkadir Özgür, Metin Çeliker, Kamil Konur

**Affiliations:** 1 Department of Internal Medicine, Faculty of Medicine, Recep Tayyip Erdoğan University, Rize 53100, Turkey; kamil.konur@erdogan.edu.tr; 2 Department of Chest Diseases, Faculty of Medicine, Recep Tayyip Erdoğan University, Rize 53100, Turkey; songul.ozyurt@erdogan.edu.tr (S.Ö.); unal.sahin@erdogan.edu.tr (Ü.Ş.); 3 Department of Otolaryngology, Faculty of Medicine, Biruni University, Istanbul 34295, Turkey; mustafatstn@gmail.com; 4 Department of Radiology, Faculty of Medicine, Recep Tayyip Erdoğan University, Rize 53100, Turkey; fatma.bceliker@erdogan.edu.tr (F.B.Ç.); mehmet.beyazal@erdogan.edu.tr (M.B.); 5 Liv Hospital, Istanbul 34340, Turkey; abdulkadir.ozgur@erdogan.edu.tr; 6 Department of Otolaryngology, Faculty of Medicine, Recep Tayyip Erdoğan University, Rize 53100, Turkey; metin.celiker@erdogan.edu.tr

**Keywords:** obstructive sleep apnea, sarcopenia, CT imaging, muscle thickness, subcutaneous fat, sarcopenic obesity

## Abstract

**Background:** Obstructive sleep apnea syndrome (OSAS) is associated with altered body composition, including increased fat accumulation and potential reductions in muscle quality. Sarcopenic obesity—a condition marked by simultaneous muscle degradation and adiposity—is of growing concern in OSAS populations. **Methods:** We conducted a retrospective study evaluating thoracoabdominal CT scans of 71 OSAS patients and 34 age- and sex-matched controls. Erector spinae muscle thickness and subcutaneous fat were measured at the L1 vertebral level. Associations with clinical markers such as BMI, CRP, and oxygen saturation were examined. **Results:** OSAS patients had significantly greater muscle thickness and subcutaneous fat compared to controls (*p* < 0.01). Muscle thickness was positively correlated with BMI and waist circumference but inversely associated with CRP and oxygen saturation. Despite structural hypertrophy, these findings suggest impaired muscle quality and support the presence of sarcopenic obesity in OSAS. **Conclusions:** CT-based imaging provides valuable structural insights but may overestimate muscle function, particularly in obese OSAS patients. These results highlight the need to integrate imaging with functional assessments to accurately diagnose sarcopenia and guide individualized management strategies.

## 1. Introduction

Obstructive sleep apnea syndrome (OSAS) is a prevalent sleep-related breathing disorder characterized by partial or complete upper airway obstruction during sleep, resulting in episodes of apnea and hypopnea. These events lead to intermittent hypoxemia and sleep fragmentation, contributing to increased cardiovascular and metabolic morbidity and mortality worldwide [1].

It is estimated that over 936 million individuals globally are affected by OSAS, with disease severity influenced by age, sex, obesity, and genetic predisposition [2]. Given the high prevalence and the fact that many individuals remain undiagnosed, there is a growing need for reliable and accessible screening tools, including novel imaging and molecular biomarkers, to facilitate early diagnosis and risk stratification [3].

Sarcopenia is a progressive disorder involving the loss of skeletal muscle strength, mass, and quality, primarily observed in older adults. When accompanied by excess adiposity, this condition is referred to as sarcopenic obesity—a multifactorial state resulting from aging, physical inactivity, hormonal imbalance, and chronic inflammation. This dual condition has been associated with impaired physical performance, increased cardiometabolic risk, and reduced quality of life [4,5].

Recent evidence suggests that OSAS may contribute to alterations in body composition similar to those observed in sarcopenic obesity. Chronic intermittent hypoxia, systemic inflammation, and neuroendocrine dysfunction observed in OSAS can impair muscle protein synthesis and promote intramuscular fat infiltration [6,7,8]. Additionally, insulin resistance and dysregulated adipokine secretion—such as elevated leptin and reduced adiponectin—may further compromise muscle quality, resulting in preserved or increased muscle size but diminished function, a phenomenon known as pseudohypertrophy [9].

In this study, we aimed to describe structural characteristics associated with muscle and fat alterations in OSAS, without asserting a diagnostic claim.

Sarcopenic obesity has also been examined in other chronic conditions such as chronic obstructive pulmonary disease (COPD) and type 2 diabetes, where combining structural imaging with functional performance tests has shown diagnostic and prognostic value [10,11]. In COPD, reduced psoas and paraspinal muscle areas on CT have been associated with decreased physical capacity and increased clinical risk [12]. Similarly, in type 2 diabetes, combining imaging methods such as CT or dual-energy X-ray absorptiometry (DXA) with objective muscle performance tests has improved the detection of functional sarcopenia and risk prediction for adverse outcomes [13].

Although prior studies often target the psoas or quadriceps muscles, the erector spinae muscle provides a practical and reproducible alternative due to its consistent visibility on thoracic and abdominal CT scans. Furthermore, recent studies have demonstrated that measurements at the T12–L1 level correlate strongly with total skeletal muscle mass and metabolic status, supporting its use in both chest and abdominal imaging protocols [14]. By analyzing erector spinae muscle and subcutaneous adipose tissue via CT at the L1 level, this study aims to provide structural insights into muscle–fat distribution patterns in OSAS patients that may be suggestive of early sarcopenic changes, without assigning a clinical diagnosis of sarcopenic obesity.

## 2. Materials and Methods

This retrospective observational study included 71 patients diagnosed with obstructive sleep apnea syndrome (OSAS) and 34 age- and sex-matched healthy individuals as controls. Participants were consecutively enrolled from the sleep disorders and radiology units of Recep Tayyip Erdoğan University Faculty of Medicine between January 2022 and December 2024. Written informed consent was obtained from all subjects. The study protocol was approved by the Institutional Ethics Committee (Approval ID: 2024/315, dated 26 December 2024), and all procedures conformed to the Declaration of Helsinki.

Inclusion criteria were as follows: (1) age ≥ 18 years; (2) availability of thoracoabdominal computed tomography (CT) scans performed within three months of polysomnography; and (3) complete clinical and laboratory data. OSAS was defined by an apnea–hypopnea index (AHI) ≥ 5 based on overnight polysomnography. Controls had AHI < 5 and no known history of sleep, metabolic, neuromuscular, or inflammatory disorders. Subjects receiving corticosteroid treatment or with a diagnosis of malignancy were excluded.

CT scans were performed using a 64-slice multidetector CT scanner (GE Healthcare, Optima CT660) in the supine position during end-expiratory breath-holding. Scan parameters were 120 kVp and 100–200 mA, with 5 mm slice thickness reconstructed using standard soft tissue algorithms. The L1 vertebral level was selected for muscle and fat measurements due to its previously demonstrated correlation with whole-body skeletal muscle mass and its consistent visibility on routine thoracic and abdominal imaging.

DICOM images were evaluated using institutional PACS software(Advantage Workstation 2.0; GE Healthcare, Waukesha, WI, USA). Bilateral erector spinae muscle thickness was measured in the anteroposterior axis at the L1 level and recorded as E.S.AP.R (right) and E.S.AP.L (left). Subcutaneous adipose tissue thickness, waist circumference, and neck circumference were measured at the same level. Measurements were independently performed by two board-certified radiologists blinded to clinical data. Inter-rater agreement was excellent, with intraclass correlation coefficients (ICC) exceeding 0.90.

To evaluate symmetry and agreement, Bland–Altman plots were generated for E.S.AP.R and E.S.AP.L values. Correlation analyses were performed between muscle thickness and clinical parameters including BMI, minimum oxygen saturation, CRP, total cholesterol, and AHI using Pearson’s or Spearman’s correlation coefficients, depending on data distribution.

Statistical analyses were performed using IBM SPSS Statistics version 25.0 (IBM Corp., Armonk, NY, USA). Normality was assessed using the Shapiro–Wilk test. Group comparisons were conducted using independent samples *t*-tests or Mann–Whitney U tests. One-way ANOVA and ANCOVA were used to compare imaging outcomes while adjusting for BMI. Correlation strengths were categorized as weak (r < 0.3), moderate (r = 0.3–0.7), or strong (r > 0.7). A two-sided *p*-value <0.05 was considered statistically significant.

Although a formal a priori power analysis was not performed, post hoc evaluation indicated that the study had sufficient power (>80%) to detect moderate effect sizes (Cohen’s d = 0.5) between groups at a significance level of 0.05, given the total sample size of 105 participants.

## 3. Results

A total of 105 participants were included in the study, comprising 71 patients with obstructive sleep apnea syndrome (OSAS group) and 34 healthy individuals (control group). Demographic characteristics and imaging measurements for each group are summarized in Table 1. The OSAS group had a slightly higher mean age (49.4 ± 10.7 years) than the control group (45.5 ± 11.7 years), and sex distribution was more balanced in the OSAS group (34 females, 37 males), whereas the control group consisted predominantly of female participants (26 females, 8 males).

Significant group differences were observed in key anthropometric and morphological parameters. The OSAS group exhibited a significantly higher body mass index (33.1 ± 5.38 kg/m^2^) compared to the control group (28.3 ± 2.38 kg/m^2^; *p* < 0.001). Furthermore, both right and left erector spinae muscle thicknesses were significantly greater in OSAS patients than in controls (E.S.AP.R: 40.5 ± 6.41 mm vs. 36.7 ± 6.2 mm, *p* = 0.005; E.S.AP.L: 41.1 ± 6.43 mm vs. 37.0 ± 6.26 mm, *p* = 0.003). Subcutaneous adipose tissue thickness was also notably higher in the OSAS group (15.3 ± 5.98 mm) compared to controls (11.0 ± 5.14 mm; *p* < 0.001). These results suggest distinct structural differences in paraspinal musculature and fat distribution associated with OSAS (Table 2).

**Figure 1:**
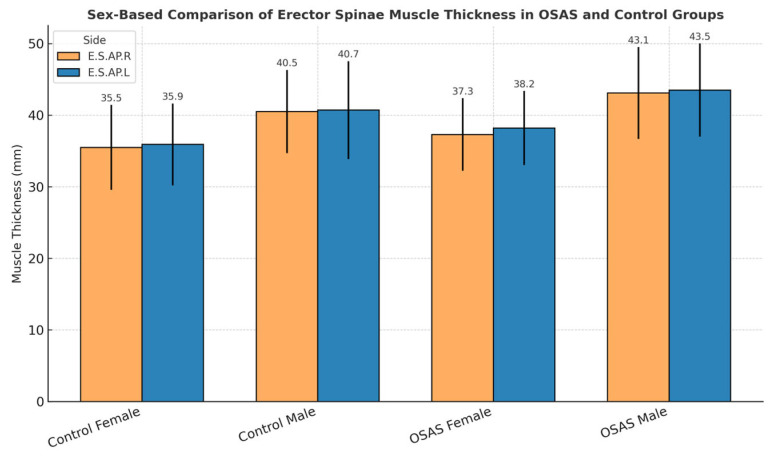
Sex-based comparison of erector spinae muscle thickness in OSAS and control groups.

Sex-based differences in erector spinae muscle thickness were observed across both diagnostic groups. As shown in Figure 1, male participants tended to exhibit greater muscle thickness than female participants for both the right (E.S.AP.R) and left (E.S.AP.L) sides in both OSAS and control groups. Although these differences did not reach statistical significance (*p* > 0.05), the consistent pattern suggests possible physiological or anatomical determinants of muscle morphology.

**Figure 2:**
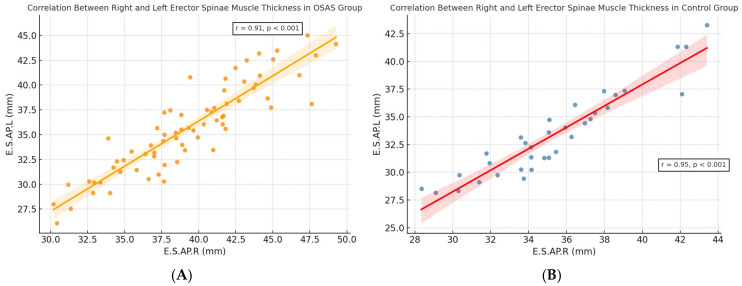
(**A**,**B**) Scatter plots showing the correlation between right (E.S.AP.R) and left (E.S.AP.L) erector spinae muscle thickness in the (**A**) OSAS group and (**B**) control group. Strong bilateral symmetry was observed in both groups (OSAS: r = 0.93, *p* < 0.001; Control: r = 0.96, *p* < 0.001).

A strong positive correlation was observed between right and left erector spinae muscle thickness in both diagnostic groups. As shown in Figure 2A,B, this bilateral symmetry was evident in OSAS (r = 0.93, *p* < 0.001) and control (r = 0.96, *p* < 0.001) groups, respectively. These findings validate the consistency of CT-based measurements and support the potential use of unilateral assessments in future morphometric evaluations.

**Figure 3:**
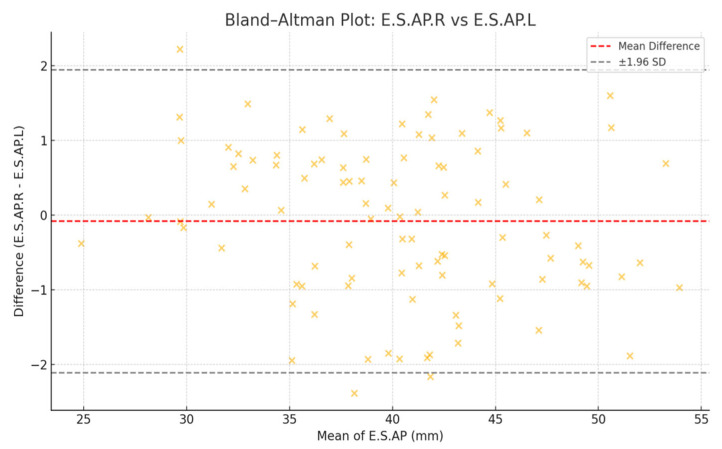
Bland–Altman plot illustrating the agreement between right and left muscle thickness measurements. Most data points lie within the 95% limits of agreement, supporting measurement reliability.

Bland–Altman analysis confirmed agreement between right and left muscle thickness, indicating high consistency between bilateral measurements (Figure 3).

**Figure 4:**
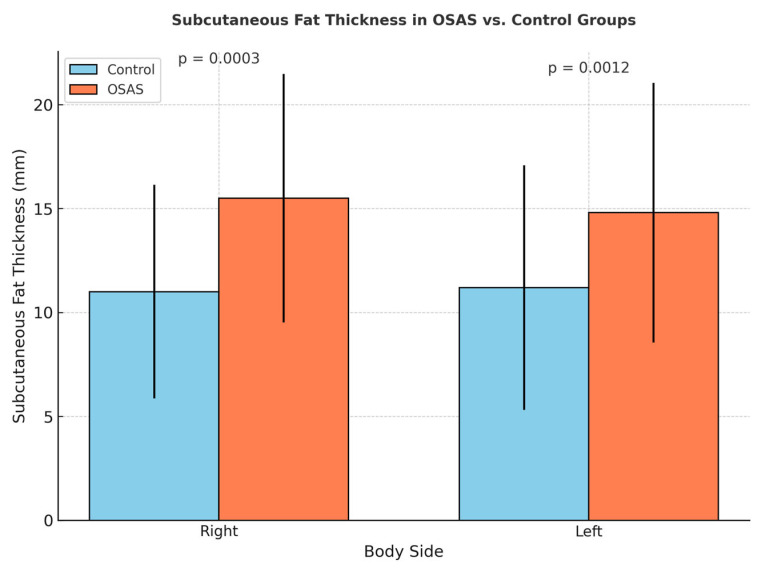
Comparison of right and left subcutaneous fat tissue thickness between OSAS and control groups.

**Figure 5:**
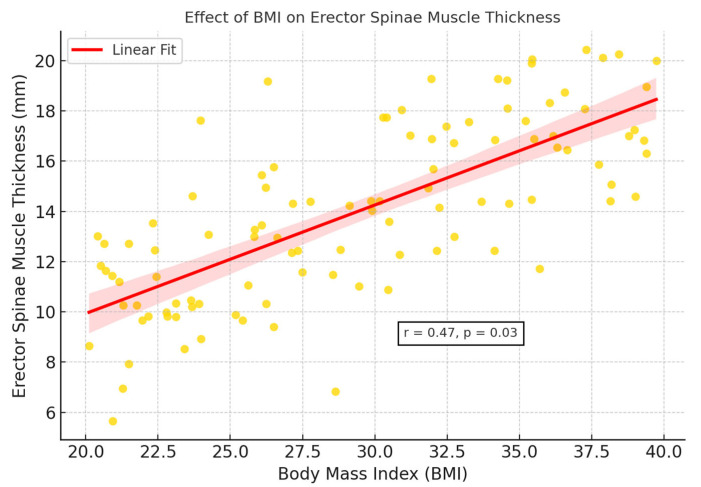
Effect of BMI on erector spinae muscle thickness.

Right and left subcutaneous fat tissue thicknesses were found to be significantly higher in the OSAS group compared to the control group (*p* = 0.0003, *p* = 0.0012) (Figure 4). This finding suggests that, despite increased muscle thickness associated with abdominal fat accumulation in individuals with OSAS, the muscle structure may reflect reduced muscle quality potentially associated with early structural features of sarcopenic obesity, although no formal functional assessment was conducted. Additionally, BMI showed a significant covariant effect on muscle thickness (*p* = 0.03) (Figure 5).

**Figure 6:**
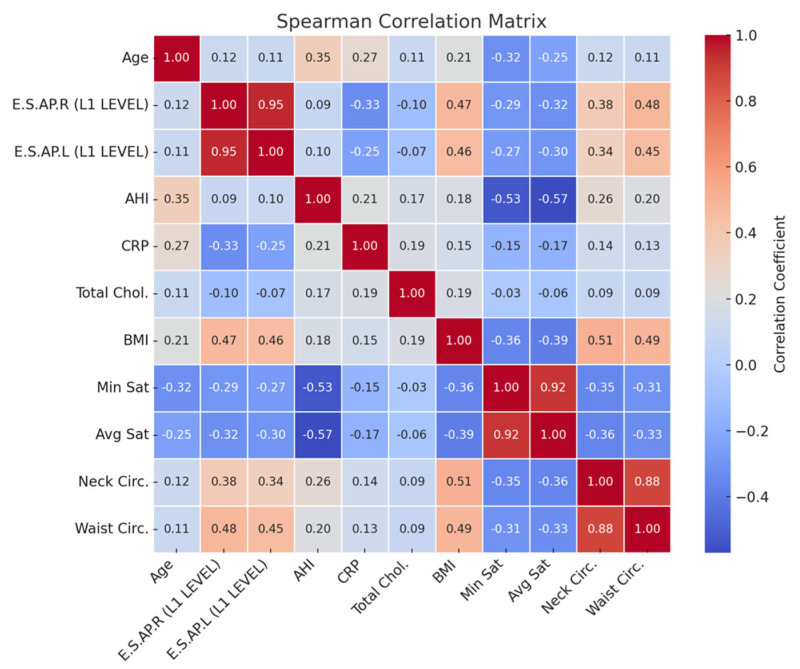
Spearman’s correlation matrix showing associations between muscle thickness and clinical parameters. Positive correlations are shown in red and negative correlations in blue, with stronger associations indicated by darker shades.

ANCOVA analysis demonstrated that BMI significantly influenced erector spinae muscle thickness (*p* = 0.03). As summarized in Table 3 and illustrated in Figure 5, BMI and waist circumference showed moderate positive correlations with muscle thickness (r = 0.45–0.48), whereas CRP and oxygen saturation indices (minimum and average) demonstrated negative correlations (r = −0.25 to −0.33). These findings suggest that the observed increases in muscle thickness among OSAS patients may be partly driven by adipose tissue infiltration, rather than reflecting true gains in muscle function. This phenomenon is consistent with the concept of pseudohypertrophy and emphasizes the need for combining morphological evaluation with functional testing in future studies to better characterize muscle quality impairments in OSAS populations (Figure 6).

## 4. Discussion

This study demonstrated that individuals with obstructive sleep apnea syndrome (OSAS) exhibit significantly increased erector spinae muscle thickness and subcutaneous adipose tissue (SAT) compared to healthy controls. However, this morphological increase in muscle size may not reflect enhanced muscle function. Instead, it may represent a phenotype characterized by muscle hypertrophy accompanied by reduced muscle quality, potentially due to intramuscular fat infiltration. Such a pattern aligns with the concept of sarcopenic obesity, in which increased lean mass coexists with functional decline. These findings underscore the need to incorporate objective functional assessments—such as grip strength or gait speed—when evaluating sarcopenia risk in OSAS populations [15].

The increased muscle thickness observed in OSAS patients may not necessarily reflect improved muscle function. Pathophysiological mechanisms such as chronic intermittent hypoxia, systemic inflammation, and hormonal dysregulation have been shown to impair mitochondrial activity and muscle regeneration while promoting the infiltration of adipose tissue within muscle fibers. This process may result in increased muscle volume without corresponding gains in functional strength—a phenomenon known as pseudohypertrophy. These mechanisms provide a plausible explanation for the dissociation between muscle size and quality observed in our findings and support the interpretation that structural measurements alone may overestimate muscle health in OSAS populations [16].

Our correlation analysis revealed that erector spinae muscle thickness was moderately positively associated with BMI and waist circumference—both indicators of central adiposity. In contrast, muscle thickness showed negative correlations with C-reactive protein (CRP) levels and minimum oxygen saturation, suggesting that systemic inflammation and hypoxemia in OSAS may compromise muscle integrity. These findings align with prior experimental studies demonstrating that intermittent hypoxia and inflammatory stress promote skeletal muscle atrophy and reduce muscle quality, independent of muscle size [17].

The strong correlation between right and left erector spinae muscle measurements (r = 0.93–0.96) and the favorable Bland–Altman agreement observed in this study confirm the anatomical consistency and reproducibility of CT-based muscle evaluation. However, relying solely on morphological data may overestimate true muscle function, especially in obese individuals, where fat infiltration can mimic hypertrophy—a phenomenon referred to as pseudohypertrophy [18].

Clinically, these findings suggest that routine CT evaluations in OSAS patients—especially those with elevated BMI—should be interpreted with caution when assessing musculoskeletal health. The structural enlargement of paraspinal muscles may mask underlying functional impairments, potentially leading to the misinterpretation of muscle integrity. Therefore, incorporating objective functional tests such as grip strength, chair–stand performance, or gait speed is essential for a comprehensive evaluation of sarcopenia, as recommended by current clinical guidelines [4].

In addition, the possibility that muscle impairments in OSAS are at least partially reversible opens avenues for therapeutic interventions. Continuous positive airway pressure (CPAP) therapy has been shown to improve systemic oxygenation and reduce inflammation, while resistance training and nutritional support may further enhance muscle quality. Longitudinal studies are warranted to determine whether such interventions can reverse or prevent muscle degradation associated with OSAS [19]. However, the absence of direct measures of muscle strength or physical performance limits definitive conclusions, and future studies should incorporate functional assessments to complement imaging findings.

Advances in imaging technologies such as radiomics and machine learning may further improve the diagnostic value of CT by enabling the detailed quantification of muscle texture and intramuscular fat content. These tools offer the potential to differentiate between metabolically active muscle tissue and non-contractile fatty infiltration, thereby providing a more precise assessment of muscle quality and aiding in risk stratification for muscle-related metabolic impairments in OSAS populations [20].

This study has several limitations. First, its retrospective design limits causal inferences and does not allow assessment of treatment effects over time. Second, although we identified structural alterations in paraspinal muscles using CT, we did not include direct measures of muscle function such as grip strength, gait speed, or chair–stand tests. Therefore, we cannot determine whether the observed muscle changes reflect preserved function or possible declines in muscle quality. Third, the absence of follow-up data restricts our ability to evaluate temporal changes or the impact of interventions. Additionally, the relatively small and sex-imbalanced sample may reduce statistical power and limit subgroup analyses by age, sex, or disease severity. Future prospective, multicenter studies combining imaging-based structural evaluation with validated functional assessments are needed to further explore the clinical implications of these findings in patients with OSAS.

Moreover, the anatomical region selected for assessment—the L1 vertebral level—offers a reproducible estimate of total skeletal muscle area but lacks direct functional interpretation. Although L3 is often considered the standard level for estimating whole-body muscle mass, L1 has demonstrated comparable prognostic value and is more frequently captured in routine thoracoabdominal imaging protocols. To enhance diagnostic precision in future research, complementary modalities such as dual-energy X-ray absorptiometry (DXA) or bioimpedance analysis (BIA) could also be considered when evaluating musculoskeletal changes in OSAS populations [21].

In OSAS patients, chronic nocturnal hypoxia triggers a cascade of metabolic changes, including the activation of hypoxia-inducible factor (HIF) pathways. These molecular responses may influence muscle fiber composition and impair oxidative metabolism, leading to reduced endurance capacity despite preserved or increased muscle mass. Translational evidence suggests that intermittent hypoxia in OSAS may suppress satellite cell activation and mitochondrial biogenesis, contributing to a mismatch between muscle structure and function [22].

Furthermore, frequent sleep fragmentation in OSAS contributes to systemic catabolism. Interrupted sleep has been shown to elevate cortisol levels and disrupt the circadian regulation of anabolic signaling pathways, which are critical for muscle protein synthesis and recovery. These disruptions may exacerbate muscle loss and favor fat accumulation within muscle tissue, particularly in older or comorbid individuals [23]. These observations highlight the need to integrate sleep quality metrics into sarcopenia-related risk models in OSAS populations.

Another area of clinical interest involves the longitudinal monitoring of muscle composition in OSAS patients receiving continuous positive airway pressure (CPAP) therapy. While previous studies have demonstrated improvements in metabolic parameters and reductions in visceral adiposity with CPAP adherence, it remains unclear whether these benefits extend to skeletal muscle architecture. Specifically, it is unknown whether CT-derived muscle quality parameters improve over time, highlighting a need for prospective trials to explore this potential therapeutic effect [24].

The observed mismatch between muscle size and function in OSAS highlights the value of integrating morphological data with objective strength testing (e.g., isometric dynamometry) and biochemical markers (e.g., creatine kinase, myostatin levels) in future work. Such multimodal assessment may refine risk stratification, guide rehabilitation protocols, and personalize OSAS management in the context of musculoskeletal health. Prospective studies utilizing such integrative methods could help establish muscle quality benchmarks and intervention thresholds tailored to OSAS severity and comorbidity burden.

## 5. Conclusions

This study demonstrates that patients with obstructive sleep apnea syndrome (OSAS) exhibit increased erector spinae muscle thickness and subcutaneous adipose tissue compared to healthy individuals. However, this structural enlargement may reflect fat infiltration and impaired muscle quality rather than functional strength, particularly in the context of central obesity.

These findings underscore that computed tomography (CT)-based morphological measurements alone are insufficient for assessing sarcopenia in OSAS. Without the concurrent evaluation of muscle strength or physical performance, structural indicators may lead to misclassification and suboptimal clinical decisions.

Clinicians should recognize that muscle hypertrophy observed on imaging in OSAS does not equate to functional integrity. Integrating imaging with functional tests (e.g., grip strength, gait speed) and biochemical markers may enable the earlier detection of muscle dysfunction and the better stratification of cardiometabolic risk.

A multimodal approach to musculoskeletal assessment is needed to guide personalized treatment strategies. Future prospective studies should evaluate how interventions such as CPAP therapy and resistance exercise affect both muscle structure and function over time in OSAS populations.

## Figures and Tables

**Table 1:** Demographic data of the patients.

Group	n	Female	Male	Mean Age	Age_SD	VKI_Ort	BMI_SD	Mean ESAPR	ESAPR_SD	Mean ESAPL	ESAPL_SD
control	34	26	8	45.5	11.7	28.3	2.38	36.7	6.2	37.0	6.26
OSAS	71	34	37	49.4	10.7	33.1	5.38	40.5	6.41	41.1	6.43

**Table 2:** Demographic and muscle thickness characteristics.

Group	Age (Mean ± SD)	BMI (Mean ± SD)	E.S.AP.R (Mean ± SD)	E.S.AP.L (Mean ± SD)	Subcutaneous Fat (Mean ± SD)	*p*-Value
Control	45.5 ± 11.7	28.3 ± 2.38	36.7 ± 6.2	37.0 ± 6.26	11.0 ± 5.14	—
OSAS	49.4 ± 10.7	33.1 ± 5.38	40.5 ± 6.41	41.1 ± 6.43	15.3 ± 5.98	<0.001 (BMI); 0.005 (E.S.AP.R); 0.003 (E.S.AP.L); <0.001 (Fat)

**Table 3:** Summary of key correlations between anthropometric, inflammatory, and oxygenation parameters and erector spinae muscle thickness.

Variable	E.S.AP.R Correlation (r)	E.S.AP.L Correlation (r)
BMI	0.47	0.46
Waist Circumference	0.48	0.45
Min Sat	−0.29	−0.27
Avg Sat	−0.32	−0.30
CRP	−0.33	−0.25

## Data Availability

The original contributions presented in this study are included in the article. Further inquiries can be directed to the corresponding author(s).
